# Calcified amorphous tumor located on a severely calcified mitral annulus in a patient with normal renal function

**DOI:** 10.1093/jscr/rjab608

**Published:** 2022-01-21

**Authors:** Ryohei Ushioda, Tomonori Shirasaka, Shinsuke Kikuchi, Hiroyuki Kamiya, Taro Kanamori

## Abstract

A calcified amorphous tumor (CAT) of the heart is a rare, nonneoplastic, intracavitary cardiac mass. Histological examination shows that it contains calcified and amorphous fibrous material with underlying chronic inflammation. Surgical excision is generally recommended to avoid future embolism. The risk of embolism has been reported to be especially high in mitral-annular-calcification-related CAT, which constitutes a subgroup of CAT that is often associated with end-stage renal disease. A case of a CAT attached to the anterior annulus of the mitral valve that was easily removed with a light touch of the forceps through aortotomy is reported.

## INTRODUCTION

A cardiac calcified amorphous tumor (CAT) is a nonneoplastic, cardiac mass composed of calcified nodules in an amorphous background of fibrin with degeneration and focal chronic inflammation [1, 2]. Since CAT can cause fatal embolism, early surgical treatment is generally recommended [4]. However, the best surgical approach, with or without simultaneous valvular intervention, has remained unclear due to its rarity and limited reports.

## CASE REPORT

**Figure 1:**
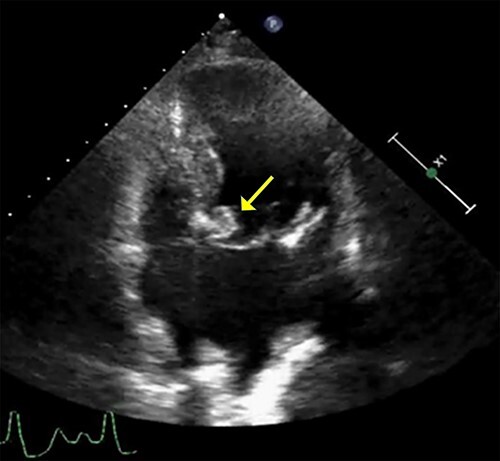
Transthoracic echocardiogram. A well-defined densely calcified mass noted on the anterior mitral leaflet in the apical four-chamber view.

**Figure 2:**
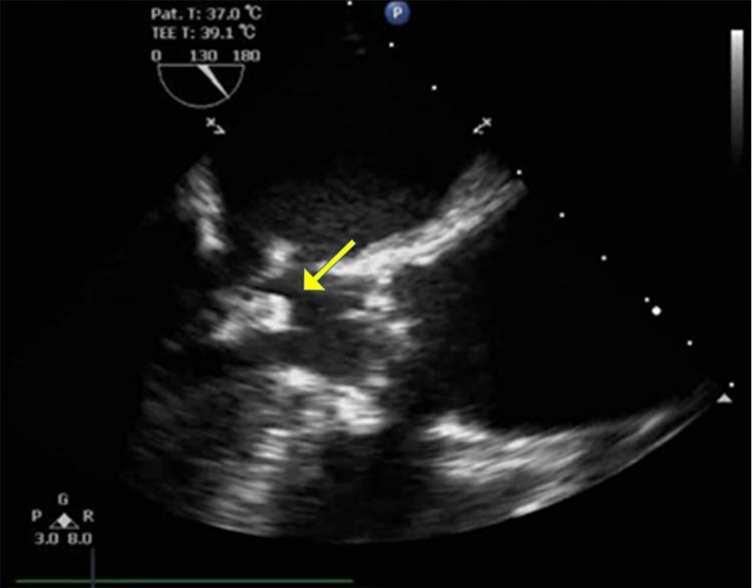
TEE in the left ventricular outflow tract view shows an echo-dense mass measuring ~12.8 mm × 12.9 mm arising from mitral annular calcification.

**Figure 3:**
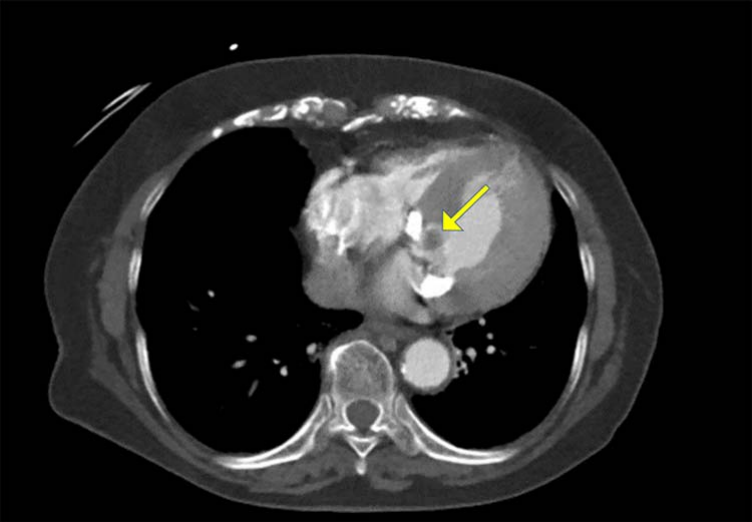
Cardiac computed tomography showing a cardiac mass in the mitral annulus with heavy mitral annular calcification.

An 86-year-old woman with a history of hypertension and hyperlipidemia presented to our hospital with episodes of syncope lasting several minutes. On admission, her consciousness was clear, and there were no significant neurological findings on physical examination. Electrocardiography showed sinus rhythm at 79 bpm. Laboratory findings showed no abnormalities in electrolytes or anemia that could cause syncope. The coronary angiogram showed 90% stenosis of the right coronary artery (RCA). Transthoracic echocardiography (TTE) and transesophageal echocardiography (TEE) both showed a mobile mass with a maximum diameter of 12 mm on the anterior annulus of the mitral valve (Figs 1 and 2). TTE showed normal left ventricle (LV) function with an ejection fraction of 60%, LV wall motion was within the normal range and no significant valvular disease was detected. Preoperative chest computed tomography showed severe mitral annular calcification (MAC) (Fig. 3).

**Figure 4:**
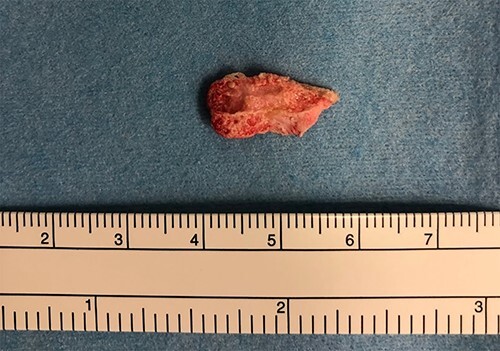
Gross appearance of the resected tumor.

**Figure 5:**
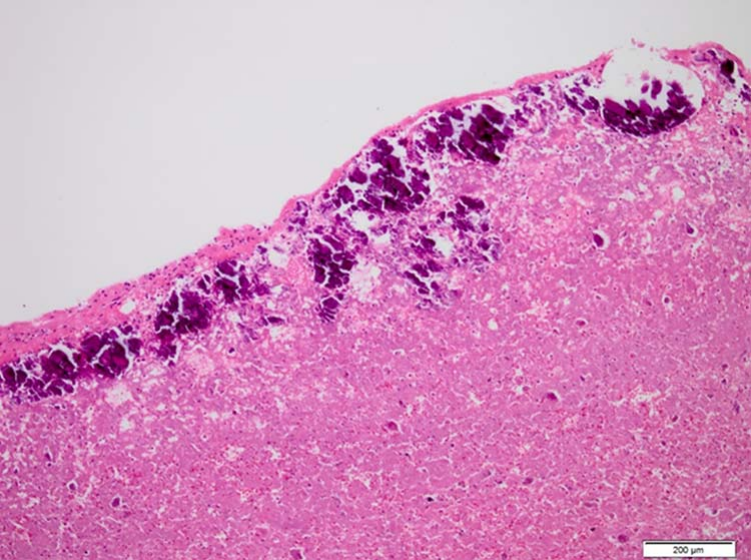
Histopathological appearance of the tumor; fine deposits of calcium surrounding the central part of the tumor, which consists of an eosinophilic muddy substance (Hematoxylin and eosin staining, ×10).

With a median sternotomy approach, cardiopulmonary bypass was established with aortic and right atrial cannulations, and cardiac arrest was induced with antegrade cold-blood cardioplegia. The tumor was easily removed with only a light touch of the forceps through aortotomy (Fig. 4). In addition, coronary artery bypass grafting (CABG) from the aorta to the posterior descending artery of the RCA with a great saphenous vein graft was also performed. The removed cardiac mass was diagnosed as a CAT on pathological examination (Fig. 5). TTE on postoperative Day 7 showed no residual lesions. On postoperative Day 10, the patient was discharged home without any complications. This patient has been followed for 1 year, and the latest postoperative TTE showed no residual tumor lesion on the mitral valve.

**Table 1:** Cases of surgery for CAT in the left heart

Report	Case No.	Age	Gender	Location of tumor	Access	Size (mm)	Surgical method	Follow-up term	Recurrence
Habib et al. (2010) [9]	1	58	M	LV,PM, MA,MV	N/A	Diffuse infiltration	resection	N/A	N/A
Kubota et al. (2010) [4]	2	64	F	LV, MA,AML	N/A	3 × 27	resection+AVR + MVR	3 years	-
	3	44	M	LV,PM,PML	N/A	6 × 27	resection	3 years	-
Greaney et al. (2011) [10]	4	69	F	LV,AML	aortotomy	20	resection	3 months	-
Ananthakrishna et al. (2011) [8]	5	45	F	LV,PML	left atriotomy	40 × 35 × 20	resection+AVR + MVR	4 months	-
Lin et al. (2011) [11]	6	74	F	atrial septum	N/A	14 × 27	resection	20 day	-
Fujiwara et al. (2012) [12]	7	58	M	MA,PML	N/A	N/A	resection+MVP	N/A	N/A
	8	65	M	LV,MA,AML	N/A	7 × 2	resection	N/A	N/A
Nazli et al. (2013) [13]	9	54	F	septoapical and anteroapical region of the LV	left ventriculotomy	38 × 25	resection	1 year	-
Yamamoto et al. (2013) [14]	10	82	F	posterior MA (P3 position)	aortotomy	37 × 4	MVR	N/A	N/A
Kawata et al. (2013) [15]	11	59	M	anterior MA	N/A	28 × 6	resection	N/A	N/A
Mohamedali et al. (2014) [5]	12	69	F	anterior MA	aortotomy	50	resection	8 days	-
Suh JH et al. (2014) [16]	13	70	F	interatrial septum	N/A	20	resection	14 months	-
Tanaka A et al. (2015) [17]	14	66	F	posterior wall of the LA	N/A	10	resection	20 days	-
de Hemptinne Q et al. (2015) [3]	15	67	M	MA, LV	aortotomy	7 × 3 × 2	resection	1 year	-
Nakashima Y et al. (2015) [18]	16	68	M	LA side of MAC	N/A	13 × 14	resection	N/A	N/A
Kinoshita M et al. (2015) [19]	17	70	M	TV, AML	N/A	diffuse infiltration	resection+MVR + TAP	N/A	N/A
Masuda S et al. (2015) [20]	18	69	F	PML (P2 and P3)	right-sided left atrial incision	19 × 8	resection+MVP	38 months	-
Abbasi Teshnizi M et al. (2017) [21]	19	37	F	LA, AML	left atriotomy	5 × 5	resection and MVP	4 days	-
Kyaw K et al. (2017) [22]	20	68	F	ventricular aspect of MV	N/A	12 × 12	resection	6 months	-
Chowdhary A et al. (2017) [23]	21	73	M	RA + LA	N/A	50 × 50,15 × 15	resection	7 months	-
Nakamaru R et al. (2017) [24]	22	70	M	PML (P2 region)	N/A	8 × 6	resection	6 months	-
Satoshi Yoshimura et al. (2017) [25]	23	64	F	PML (P2 region)	N/A	15	resection	N/A	N/A
Nozomi Toyokawa et al. (2018) [26]	24	75	F	PML	N/A	N/A	resection	N/A	N/A
Amit C. Shah et al. (2018) [27]	25	54	M	LV, PM	N/A	12 × 5	resection	8 months	-
Yoshihiro Aizawa et al. (2018) [28]	26	38	M	AML	sagittal incision of the left atrium	diffuse infiltration	MVR	10 months	-
Eroğlu M et al. (2019) [29]	27	56	F	LA, PML	right atriotomy and interatrial septotomy	20 × 30	MVR	24 months	-
Azin Alizadehasl et al. (2019) [30]	28	43	M	LA, atrial side of the MA	N/A	20 × 6	MVR	7 days	-
Michael Chetrit et al. (2020) [31]	29	77	M	posterior sife of the MA	N/A	diffuse infiltration	MVR	N/A	N/A
	30	59	M	anterolateral commissure of the MV, LV	aortotomy	16 × 5	resection	N/A	N/A
Takashi Suzue et al. (2021) [32]	31	67	M	posterior commissure of the MV	aortotomy	50 × 13	resection	1 year	-

## DISCUSSION

Cardiac CAT was first reported by Reynolds *et al*. [1] in 1997 as a nonneoplastic, intra-cardiac mass composed of calcified deposition and amorphous fibrous tissue. Histologically, it is characterized by nodular calcium deposits over a matrix of fibrin and/or amorphous fibrin-like material, hyalinization, inflammatory cells and degenerated hematological elements. It has been linked to organized thrombi, but its precise etiology is unknown [2]. In the present case, pathological findings showed a CAT with deposits of calcium surrounding the central part of the tumor, which consisted of an eosinophilic muddy substance.

This tumor can originate in any of the four cardiac chambers, but the most common site is the mitral annulus [3]. de Hemptinne Q *et al*. analyzed 42 cases of CAT and reported that CAT was detected in all cardiac chambers, but predominated on the mitral valve or annulus (36%), followed by the right atrium (21%) and the right ventricle (17%). Patients with CAT frequently present with dyspnea and syncope [3]. In the present case, the patient’s chief complaint was loss of consciousness. It is well known that the risk of embolization in patients with intracardiac CAT is very high, because the CAT is normally weakly attached to surrounding tissues [4]. Therefore, most of the time these symptoms are related to embolization or obstruction, depending on its size and location. Moreover, CAT was associated with end-stage renal disease (ESRD) in 21% of patients and MAC was found in 14% [3]. Furthermore, MAC-related CAT is usually seen in ESRD patients [4, 5]. MAC-related CAT with normal renal function, as in the present case, is very rare, and there have been only a few reported cases [3, 6].

A systematic search of PubMed was performed to identify articles reporting cases of CAT in the English language literature. All articles published since the first report (1 May 1997) up to 31 August 2021 were included. The titles and abstract of the identified articles were screened to determine if they met inclusion criteria. Full-text articles were then retrieved and reviewed. Reference lists of the retrieved articles were searched for relevant literature. The review yielded 31 cases from 28 reports. Surgical resection of CAT is usually easy when adequate exposure of the tumor is achieved. The case reports were reviewed in relation to the surgical resection of CAT, and it was found that it seems possible to access the LV cavity and resect it all through the aortic valve with aortotomy. Therefore, no special approach is required, and valve replacement should be performed only if the CAT is very large causing valve obstruction, or if there is pre-existing valve disease [7, 8]. In the present case, the CAT was easily removed just by a light touch of the forceps, meaning that the risk of embolism would be extremely high if left untreated. Regarding the approach between aortotomy and left atriotomy, from the experience in the present case, aortotomy could be a better choice for CAT if it is on the left ventricular aspect of the anterior mitral leaflet. In addition, as mentioned above, MAC-related CAT is associated with impaired renal function, so shortening the hypoperfusion time of the kidneys is important to prevent further deterioration of kidney function. When there is renal dysfunction or the patient is elderly, simple procedures such as aortotomy could be a better choice.

Recurrence of CAT seems quite rare. There were no reported CAT recurrences during a mean follow-up of 13.5 months among the 31 reviewed cases (Table 1). In addition, only a single case [2] reported recurrence of a right atrial CAT 3 years after surgery (not shown in Table 1). Therefore, in our institution, follow-up is performed once a year just in case.

## CONCLUSIONS

A case of MAC-related CAT in a patient with normal renal function was presented. Because CAT arising from MAC has been reported to be associated with a high risk of embolism, and surgical removal is normally easy without need for valve replacement, as in the present case, early surgical treatment would be recommended.

## CONFLICT OF INTEREST STATEMENT

None declared.
